# THE PROCESS OF CULTURALLY ADAPTING AND TAILORING AN EXISTING PSYCHOEDUCATION PROGRAM FOR BLACK DEMENTIA CAREGIVERS

**DOI:** 10.1093/geroni/igae098.0852

**Published:** 2024-12-31

**Authors:** Fayron Epps, Karah Alexander, Stephanie Bennett, Carolyn Clevenger, Kenneth Hebpurn

**Affiliations:** The University of Texas Health San Antonio, San Antonio, Texas, United States; Emory University, Atlanta, Georgia, United States; Emory University, Atlanta, Georgia, United States; Emory University, Atlanta, Georgia, United States; Emory University, Atlanta, Georgia, United States

## Abstract

Racial differences and disparities related to caregiving experiences exist at alarming rates for service utilization, care hours, and living below the federal poverty level among Black Americans compared to White caregivers. We developed a course, Caregiving while Black, to appreciate the culturally salient contexts, values, roadblocks, and education for Black caregivers of persons living with dementia. This project moved beyond existing psychoeducational interventions focusing mainly on dementia-related or crisis management education for family caregivers by keeping equity issues at the forefront of caregiver education. The course took an assertively “For Us; By Us” approach, prominently employing the voices and faces of Black healthcare providers, family caregivers, and PLWD. Building on materials from another caregiver psychoeducation crisis management course, we adapted and tested the course in a no-control longitudinal trial (n =75). This was a time-consuming process that took approximately 12 months. We learned that community partners, providers, and caregivers were eager to be involved in the course’s design. Out of the 75 caregivers who consented, 32 completed the course. Caregivers who couldn’t complete the course indicated they lacked motivation, wanted more guidance, and desired face-to-face interaction. Several Black faith communities echoed the preference for face-to-face interaction. This feedback will direct the revision of the Caregiving while Black course by offering greater interactivity and a cohort structure with a synchronous component to complement the asynchronous content to add greater interactivity. Overall, Black caregivers greatly appreciated the team designing a course for them.

